# Relationship of Dickkopf1 (DKK1) with Cardiovascular Disease and Bone Metabolism in Caucasian Type 2 Diabetes Mellitus

**DOI:** 10.1371/journal.pone.0111703

**Published:** 2014-11-04

**Authors:** Antonia Garcia-Martín, Rebeca Reyes-Garcia, Beatriz García-Fontana, Sonia Morales-Santana, Ana Coto-Montes, Manuel Muñoz-Garach, Pedro Rozas-Moreno, Manuel Muñoz-Torres

**Affiliations:** 1 Bone Metabolic Unit (RETICEF), Endocrinology Division, Hospital Universitario San Cecilio, Instituto de Investigación Biosanitaria de Granada, Granada, Spain; 2 Endocrinology, Hospital Comarcal del Noroeste, Caravaca de la Cruz, Murcia, Spain; 3 Endocrinology Unit, Hospital General Universitario Rafael Mendez, Lorca, Murcia, Spain; 4 Proteomic Research Service, Fundación para la Investigación Biosanitaria de Andalucía Oriental -Alejandro Otero- (FIBAO), Granada, Spain; 5 Department of Morphology and Cellular Biology (RETICEF), Faculty of Medicine, University of Oviedo, Oviedo, Spain; 6 Critical Care and Emergencies Unit, Hospital Universitario San Cecilio, Granada, Spain; 7 Endocrinology Division, Hospital General de Ciudad Real, Ciudad Real, Spain; Medical University Innsbruck, Austria

## Abstract

**Objectives:**

Dickkopf-1 (DKK1) is a potent inhibitor of Wnt signalling, which exerts anabolic effects on bone and also takes part in the regulation of vascular cells. Our aims were to evaluate serum DKK1 in type 2 diabetes (T2DM) patients and to analyze its relationships with cardiovascular disease (CVD). We also evaluated the relationship between DKK1 and bone metabolism.

**Design:**

We conducted a cross-sectional study in which we measured serum DKK1 (ELISA, Biomedica) in 126 subjects: 72 patients with T2DM and 54 non-diabetic subjects. We analysed its relationship with clinical CVD, preclinical CVD expressed as carotid intima media thickness (IMT), and bone metabolism.

**Results:**

T2DM patients with CVD (*P* = 0,026) and abnormal carotid IMT (*P* = 0,038) had higher DKK1 concentrations. DKK1 was related to the presence of CVD in T2DM, independently of the presence of risk factors for atherosclerosis. Therefore, for each increase of 28 pg/ml of serum DKK1 there was a 6,2% increase in the risk of CVD in T2DM patients. The ROC curve analysis to evaluate the usefulness of DKK1 as a marker for high risk of CVD showed an area under the curve of 0,667 (95% CI: 0,538–0,795; *P* = 0,016). In addition, there was a positive correlation between serum DKK1 and spine bone mineral density in the total sample (r = 0,183; *P* = 0,048).

**Conclusion:**

In summary, circulating DKK1 levels are higher in T2DM with CVD and are associated with an abnormal carotid IMT in this cross-sectional study. DKK1 may be involved in vascular disease of T2DM patients.

## Introduction

Patients with type 2 diabetes mellitus (T2DM) have a higher risk of cardiovascular disease (CVD). This association has serious consequences on the morbidity and the mortality in this population. Atherosclerosis is the main pathological mechanism in macrovascular disease in T2DM, which causes the development of atheroma plaques and the major life-threatening events in atherosclerosis, including myocardial infarction and stroke [1].

Patients with osteoporosis have a higher prevalence of cardiovascular disease, and it has been proposed that both pathologies share common pathophysiologic pathways [2]. In T2DM there is an increased risk of fractures at any skeletal site even with greater bone mineral density (BMD) [3], [4], and there are data from epidemiological studies supporting a relationship between low bone mineral density (BMD) and atherosclerosis in T2DM [5], [6]. Thus, a better characterization of the pathways that could be involved in both processes is of interest, and may help in the development of new therapeutic tools.

The Wnt signalling pathways are involved in several developmental and physiological processes, including the cell differentiation and tissue/organ morphogenesis [7]. The discovery of the Wnt signaling pathway and its relevance in bone homeostasis has contributed to a better knowledge of the cellular and molecular mechanisms of bone biology [8]. The activation of this pathway results in an expansion of osteoprogenitor cells and also determines a reduction in the apoptosis of osteoblasts, which leads to anabolic effects on bone [8]. The canonical Wnt pathway is regulated by several families of secreted antagonists, such as the soluble frizzled-related receptors, dickkopf-1 (DKK1) and sclerostin. DKK1 regulates the Wnt signaling by binding to the Wnt co-receptor, the low-density lipoprotein-related receptor (LRP) 5/6. In addition to its binding to LRP 5/6, DKK1 binds to other trans-membrane molecules as proteins Kremen, which increases their inhibitory activity on the Wnt signaling pathway [9].

The role of Wnt signaling pathways in atherosclerosis it is a matter of growing interest. In preclinical studies the Wnt signaling pathways are involved in atherosclerosis-related processes as vascular calcification [10], inflammation [11], and the monocyte adhesion and its trans-endothelial migration [12]. In a recent study conducted in bovine aortic endothelial cells (ECs) [13], the authors found that Msx2 and Wnt7 family members stabilize the aortic ECs phenotype, whereas DKK1 promotes an endothelial–mesenchymal transition in cultured bovine aortic ECs, a process associated to the pathogenesis of myocardial fibrosis with ischemia [14] and valve calcification [15]. In addition, in animal models of CKD-MBD (chronic kidney disease-mineral bone disease) [16] there is an increased renal production of Wnt inhibitor family members and higher levels of circulating DKK1, sclerostin, and secreted klotho. In this model, the neutralization of DKK1 by administration of a monoclonal antibody after renal injury stimulated bone formation rates, corrected the osteodystrophy, and prevented CKD-stimulated vascular calcification. Besides, the neutralization of DKK1 suppressed aortic expression of the osteoblastic transcription factor Runx2, increased expression of vascular smooth muscle protein 22-α, and restored aortic expression of klotho”

There is also recent data showing a relationship between serum DKK1 and atherosclerosis in humans [17], [18]. In a previous study from Register and colleagues, DKK1 concentrations were inversely related to coronary artery disease and aortic calcification. However, as the authors point, this study has been conducted in Afro-Americans (AAs) with T2DM, which has a different prevalence of atherosclerotic disease and osteoporosis compared to European populations. These differences make it interesting to explore this relationship in Caucasian type 2 diabetes. Besides, the relationship between DKK1 concentrations and bone mass has been explored with conflicting data [19]–[21], so its evaluation in a population with a high risk of bone fragility such as T2DM is of interest.

In this context, the objectives of our study were to evaluate serum DKK1 levels in a cohort of T2DM patients and to analyze its relationships with CVD and bone metabolism. In addition, we compared serum DKK1 concentrations between T2DM and non-diabetic subjects. Thus, our hypothesis was that DKK1 concentrations may be related to CVD and bone metabolism in T2DM patients.

## Materials and Methods

### Study population

Our study was a cross-sectional one that included 126 subjects. The estimated size of the sample considering an error of 5% and a statistical power of 80% was 84 subjects. The T2DM group included 72 patients with a diagnosis of diabetes according to the American Diabetes Association criteria (ADA, 2005). From January 2006 to December 2007 we consecutively recruited patients who had been referred to our outpatient clinic from community clinics for treatment of diabetes. The control group included 54 non-diabetic subjects who were consecutively recruited from the general community in the same period of time.

All participants were recruited according to the following criteria: Caucasian, free-living, aged 35 to 65 years and normal values for blood count, renal creatinine, hepatic function, calcium and phosphorus. We considered as exclusion criteria the presence of chronic diseases apart from T2DM in the non-diabetic group, the presence of diseases affecting bone (Paget's disease, rheumatoid arthritis, hyperparathyroidism, hypercortisolism, malignant tumors, renal bone disease, chronic liver disease and post-transplantation bone disease) and also previous or current treatment with drugs affecting bone metabolism (calcium supplements, vitamin D preparations, selective estrogen receptor modulators, calcitonin, estrogens therapy, antiresorptive therapy, thiazides, steroids, glucocorticoids or anticonvulsants). Patients treated with thiazolidinediones were also excluded.

The presence of cardiovascular disease was recorded by the medical history and after that it was checked in medical records. T2DM patients were classified into two groups according to the presence of clinical CVD: CVD group and non-CVD group. Inclusion criteria for patients with CVD were cerebrovascular disease (ischemic stroke or transient ischemic attack); coronary heart disease (previous myocardial infarction, diagnosed stable or unstable angina, or coronary revascularization surgery) or ischemic peripheral arterial disease (diagnosed by a positive Doppler ultrasound or arteriogram, or by a previous revascularization surgery).

The study was approved by the ethical review board of the Hospital Universitario San Cecilio and it was done conformed to the ethical guidelines for research in humans. All the participants in the study provided written informed consent after a full explanation of the purpose of the study and the nature of all the procedures that will be used in the study. Written informed consent was approved for the ethical review board of our hospital, and after that it was filed in the medical record of each patient.

### Clinical evaluation

Height, weight, and waist circumference were measured at baseline according to standard procedures. Weight was measured to the nearest 100 grams using digital electronic scales. Height and waist circumference were measured to the nearest 1 mm using a stadiometer and a metal anthropometric tape, respectively. Body mass index (BMI) in Kg/m^2^ was calculated as weight divided by the square of height in meters.

Blood pressure was measured in a standardized manner. The subjects remained at rest for at least 5 minutes, and after that blood pressure was measured twice using a standard mercury sphygmomanometer (12 centimeters long, 35 cm wide). The mean of the two values was used for analysis. We defined hypertension when blood pressure values were higher than 140/90 mmHg and/or where subjects were on antihypertensive treatment.

The participants reported their alcohol use, smoking status and their level of physical activity with a specific health questionnaire. Patients were classified as having a significant alcohol intake if it was higher than 40 grams/day in males and 24 grams/day in women; smoking status was categorized as no tobacco use or current tobacco use. Physical activity was collected through a specific questionnaire in which study subjects scored their physical activity on a scale from 0 (none) to 10 (sport more than one hour four times per week). Based on the results, the study sample was divided into two groups: sedentary (<5 on the scale) and no sedentary (≥5 on the scale).

### Serum measurements

Samples of venous blood were taken in the morning after a fasting overnight. The sera were stored at −80°C until examination. Fasting plasma glucose (FPG), glycated hemoglobin (HbA1c), calcium, phosphorus and creatinine were measured using standard automated laboratory techniques. Glomerular filtration rate (GFR) was calculated by MDRD/CKD-EPI formula [22]. High-density lipoprotein (HDL), low-density lipoprotein (LDL) cholesterol and triglycerides (TGs) were measured by standard biochemical methods. Dyslipidemia was defined according to third report of the expert panel on detection, evaluation, and treatment of high blood cholesterol in adults (ATP-III) criteria or current treatment with statins.

Calciotropic hormones measurement included serum intact parathormone (Two-site immunoassay for iPTH, Roche Diagnostics SL, Barcelona, Spain; intra and interassay variability of 3%) and 25-hydroxyvitamin D (25-OH-D, radioimmunoassay, DiaSorin, Stillwater, Minnesota USA. Serum DKK1 was measured using quantitative sandwich enzyme-linked immunosorbent assay (ELISA) developed by Biomedica (Biomedica Medizinprodukte GmbH and Co. KG, Wien, Austria) according to the manufacturer's instructions. The Biomedica DKK-1 ELISA (BI-20412) detects free DKK-1. Intra-assay and interassay variability were of 7% and 9%, respectively. DKK1 measurements are reported throughout in pg/ml after conversion from pMol/L. 1 pMol/l is equivalent to 28,68 pg/ml.

### Bone density measurement and vertebral fractures assessment

Bone mineral density at lumbar spine (LS) L2–L4, femoral neck (FN) and total hip (TH) was performed in all patients by dual-energy X-ray absorptiometry (DXA) using Hologic QDR 4500 densitometer (Whatman, MA; variation coefficient <1%). All the BMD measurements were done by the same experienced operator. We used the World Health Organization criteria for osteoporosis (Kanis JA, 1994).

Standardized spinal X-rays were taken for morphometric analysis of all participants of the study and they were interpreted according to the algorithm developed by McCloskey and colleagues [23].

**Carotid intima-media thickness** Ultrasonographic examination of the carotid arteries was performed with patients in supine position using Doppler ultrasonography (Toshiba PowerVision 6000). The maximum intima-media thickness (IMT) at the carotid bifurcation (BIF) was determined between the near and far walls of the carotid bifurcation on the right and left sides. Each part was measured from views of both longitudinal and vertical sections at the bifurcation. If a discrepancy was observed in the measured values between longitudinal and vertical sections, the smaller value was selected to avoid overestimation. BIF–IMT was defined as the mean of the measurements from the right and left sides. The value for each side was obtained from the mean of 10 wall measurements. The BIF–IMT, measured in millimeters (mm), was considered pathological if it was ≥0.9 mm, and was considered to be carotid atherosclerosis if the BIF–IMT was ≥1.2 mm or 50% greater than the BIF–IMT in the adjacent area [24]. Plaques were identified as calcified by findings of bright white echoes on sonography. A single trained sonographer performed the study in all subjects.

### Statistical analysis

Data for continuous variables are presented as mean ± standard deviation (SD). Data for categorical variables are presented as numbers and/or percentages. Kolmogorov–Smirnov test was used to test the normality of distribution of continuous variables. The association between continuous variables was described by Pearson's or Spearman's correlation coefficients. The comparison of categorical variables among groups was performed using Chi-square test or Fisher test. The comparison of continuous variables among groups was performed using unpaired Student's *t* test or Mann-Whitney test. To determine the independent variables which correlated with DKK1 (dependent variable), the parameters that correlate significantly in univariate analysis and other which are biologically linked to DKK1 were tested in multiple backward model linear regression analysis. Multiple backward model of logistic regression analysis was performed to identify DKK1 as independent predictor for CVD (dependent variable) in T2DM patients. The model included established atherosclerotic risk factors (age, gender, body mass index, hypertension, dislipidaemia, smoking, sedentarism, HbA1c, GFR and intima-media thickness). CVD-defining parameters (cerebrovascular disease, coronary heart disease or ischemic peripheral arterial disease) were not included in the multiple logistic regression model. The usefulness of serum DKK1 as a marker of high risk of atherosclerotic disease in T2DM was analyzed using a receiver operating characteristic curve (ROC Curve). A p value of less than 0,05 was considered to be significant (two-tailed). Data were recorded and analyzed using SPSS version 18.0 software (SPSS Inc, Chicago, IL, USA).

## Results

**Figure 1:**
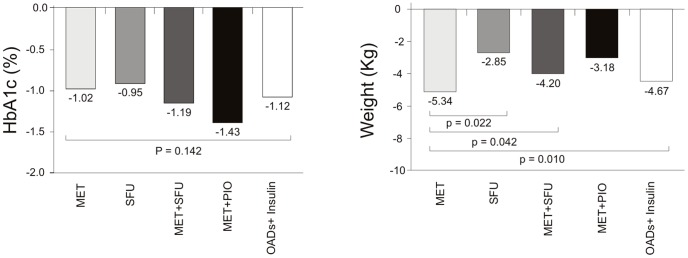
DKK1 serum levels in T2DM patients according to the presence of cardiovascular disease and abnormal intima-media thickness. Between groups differences are indicated through a bar with the P-value given above.

The clinical characteristics of the study population are summarized in Table 1. Serum DKK1 was significantly higher (*P* = 0,026) in T2DM patients with CVD (757±416 pg/ml) compared with those without CVD (547±333 pg/ml). (Figure 1 and Table 2). T2DM patients with abnormal IMT (756±433 pg/ml *vs* 563±322 pg/ml; *P* = 0,042) and cerebrovascular disease (854±480 pg/ml *vs* 625±363; *P* = 0,045) had higher DKK1 concentrations. (Figure 1 and Table 2). However, we did not find differences in DKK1 concentrations according to the presence of carotid plaque (*P* = 0,522), coronary heart disease (*P* = 0,677) or ischemic peripheral artery disease (*P* = 0,762).

**Table 1:** Characteristics of the study population.

	Total Group (n = 126)	T2DM Group (n = 72)	Non-diabetic Group (n = 54)	P Value
Age (years)	57±6	58±6	55±7	0,018
Male/female (n)	62/64	39/33	25/29	0,472
**Medical history**				
Duration of diabetes (years)	-	13,7±7,6	-	
Hypertension (%)	53,2	80,6	46,3	<0,001
Dyslipidaemia (%)	65,9	94,4	70,4	<0,001
Albuminuria (%)	20,5	35,0	4,0	0,006
**Chronic kidney disease (KDOQI stages) (%)**				
Stage 1	51	52	52	0,938
Stage 2	46	44	48	0,815
Stage 3	3	4	0	0,155
Smoker or ex-smoker (%)	15,1	16,7	13	0,623
Alcohol (%)	8,7	6,9	11,1	0,104
Sedentarism (%)	47,6	55,6	37,0	0,048
**Clinical evaluation:**				
BMI (kg/m^2^)	102,6±12,4	31,4±5,7	29,3±5,9	0,043
Waist circumference (cm)	130±20	106,4±11,4	97,4±11,9	<0,001
SBP (mm Hg)	80±13	134±97	124±17	0,002
DBP (mm Hg)	30,5±5,9	80±12	79±15	0,705
**Serum parameters:**				
FPG (mg/dL)	137,2±61,9	173±60,1	89,4±10,4	<0,001
HbA1_c_ (%)	6,7±2,2	8±1,9	4,8±0,4	<0,001
GFR (MDR/CKD-EPI)(ml/min/1,73 m^2^)	92±23	92±23	93±22	0,745
Calcium (mg/dL)	9,5±0,5	9,6±0,5	9,3±0,4	0,001
Phosphorus (mg/dL)	3,6±0,5	3,7±0,5	3,5±0,5	0,01
PTH (pg/mL)	43,6±19,5	38,5±18,4	50,4±19,1	<0,001
25(OH) D (ng/mL)	19,5±11,3	17,8±11,5	21,6±10,9	0,06
Triglyceride (mg/dl)	142±121	169,9±149,8	104,9±47,7	<0,001
HDL-c (mg/dl)	53,5±15,5	49±16	59,5±12,5	<0,001
LDL-c (mg/dl)	111,7±35,5	96,9±34,1	130,8±27,4	<0,001
DKK1 (pg/ml)	629±374	669±395	575±340	0,163
**DXA parameters and VF:**				
BMD LS (g/cm^2^)	0,977±0,148	0,954±0,146	1±0,148	0,068
BMD FN (g/cm^2^)	0,820±0,124	0,817±0,132	0,823±0,117	0,792
BMD TH (g/cm^2^)	0,906±0,135	0,903±0,145	0,911±0,125	0,772
T-score LS	−1,08±1,36	−1,3±1,3	0,82±1,3	0,058
T-score FN	−0,55±1,01	−0,6±1,04	−0,49±0,99	0,565
T-score TH	−0,55±0,98	−0,62±1	−0,51±0,92	0,557
Osteoporosis (%)	15,9	24,6	9,4	0,047
Morphometric VF (%)	23,0	30,3	20,0	0,274
**Cardiovascular disease:**	35,7	58,3	5,6	<0,001
Cerebrovascular disease (%)	11,9	19,4	1,9	0,002
Coronary heart disease (%)	23,8	38,9	3,7	<0,001
Peripheral artery disease (%)	7,9	13,9	0	0,005
Abnormal intima-media thickness (%)	35,7	54,2	11,1	<0,001
Carotid plaques (%)	15,9	29,4	0	<0,001

Data for continuous variables are presented as mean ±SD. Data for categorical variables are presented as numbers and/or percentages. The comparison between groups was done by Student's t test (continuous variables) or Chi-square test (categorical variables).

T2DM: type 2 diabetes mellitus; BMI: body mass index; SBP: sistolic blood pressure; DBP: diastolic blood pressure; FPG: fasting plasma glucose; HbA1c: glycated hemoglobin; GFR: glomerular filtration rate; MDR/CKD-EPI: Chronic Kidney Disease Epidemiology Collaboration; PTH: parathormone; 25(OH) D: 25-hydroxyvitamin D; HDL-c: High-density lipoprotein; LDL-c: Low-density lipoprotein; BMD: bone mineral density; LS: lumbar spine; FN: femoral neck; TH: total hip; VF: vertebral fractures.

**Table 2:** DKK1 concentrations according to the presence of cardiovascular disease in the T2DM group.

	Yes	No	P value
Cardiovascular disease	757±416	547±333	0,026
Cerebrovascular disease	854±480	625±363	0,045
Coronary heart disease	694±379	654±408	0,677
Peripheral artery disease	634±247	675±415	0,762
Abnormal intima-media thickness	756±433	567±322	0,042
Carotid plaque	730±422	661±395	0,522

The comparison between groups was done by Student's t test.

A model of logistic regression analysis was performed using the presence of CVD as a dependent variable. Independent variables were serum DKK1 levels and risk factors for atherosclerosis (age, gender, body mass index, hypertension, dislipidaemia, smoking, sedentarism, HbA1c, GFR, and IMT). DKK1 levels were independently associated with the presence of CVD in T2DM (odds ratio: 1,062, 95% confidence interval: 1,003–1,125; *P* = 0,04). Therefore, for each increase of 28 pg/ml of serum DKK1 there was a 6,2% increased risk of CVD in T2DM patients.

**Figure 2:**
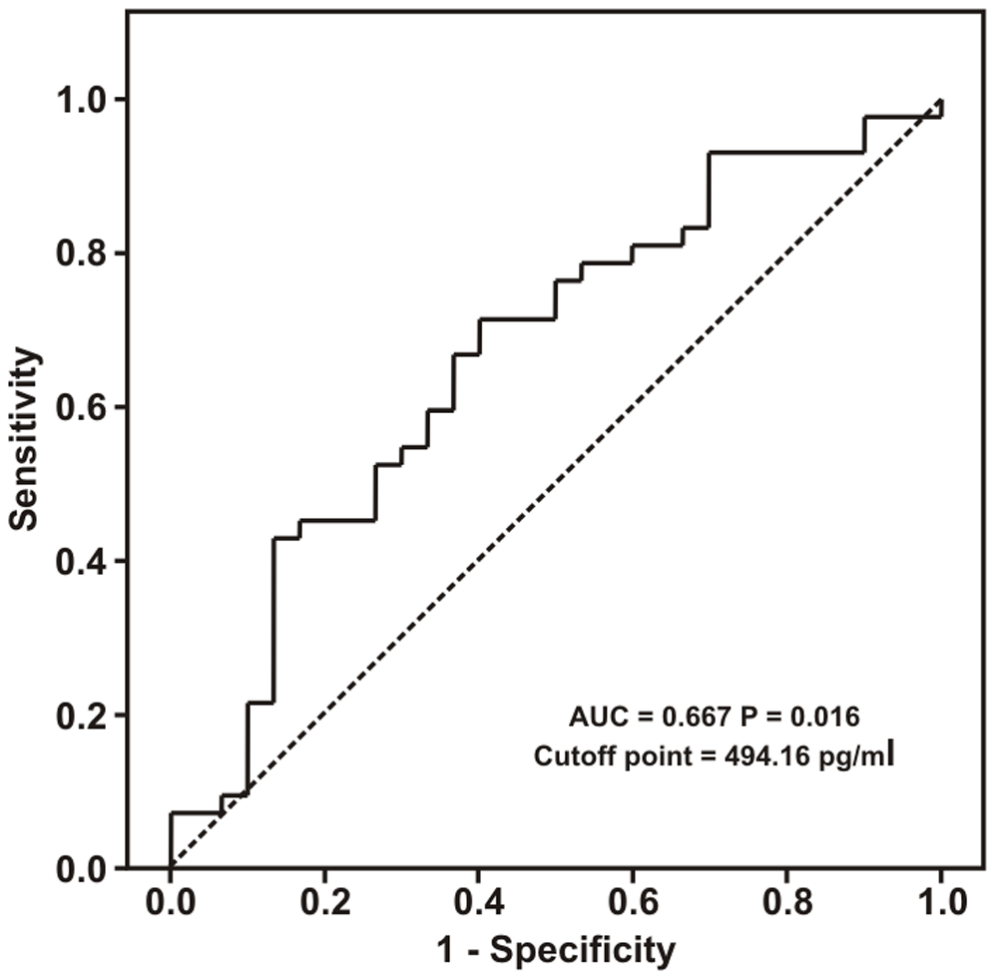
DKK1 ROC curve for cardiovascular disease.

In the ROC curve analysis to evaluate the usefulness of DKK1 as a marker for high risk of CVD, the area under the curve was 0,667 (95% confidence interval: 0,538–0,795; *P* = 0,016) (Figure 2). A concentration of 494 pg/ml or higher showed a sensitivity of 71,4% and a specificity of 60% to identify an increased risk of CVD.

We found no differences in DKK1 between study groups: T2DM 669±395 vs non-diabetic 575±340 pg/ml, *P* = 0,163. Serum DKK1 concentrations were significantly higher in females than in males in total sample (697±434 vs 563±293 pg/ml, *P* = 0,046) and in T2DM group (788±493 vs 569±255 pg/ml, *P* = 0,025) but were not significantly increased in control females (593±335 vs 553±349 pg/ml, *P* = 0,682).

Linear regression analysis was performed to determine the influence of independent factors identified in univariate correlation analysis, including group, gender, age, HbA1c, CVD and IMT as independent variables that explain serum DKK1 levels. The analysis demonstrated that gender (β = −0,299, *P* = 0,002) and CVD (β = 0,292, *P* = 0,007) were positively associated with serum DKK1.

Regarding bone metabolism, lumbar spine BMD was positively related to serum DKK1 in total sample (r = 0,183, *P* = 0,048). In contrast, we did not found a significant relationship with bone remodelling markers osteoporosis diagnosis or morphometric vertebral fractures (data not shown), in the total sample or in the study groups separately.

## Discussion

To our knowledge, there is no previous study evaluating DKK1 concentrations and its relationship with CVD in Caucasian T2DM patients. There is also scarce data concerning the relationship between DKK1 and bone metabolism in T2DM. Our results show that higher levels of DKK1 are associated with clinical CVD and abnormal IMT in T2DM patients. These findings suggest that serum DKK1 are related to the presence of CVD in this population. However, our data showed no differences in serum DKK1 between T2DM patients and non-diabetic patients. Regarding bone metabolism, we found a significant relationship with bone mineral density.

In our study, higher DKK1 levels were positively related to cardiovascular disease in T2DM patients independently of the presence of others risk factors for atherosclerosis, and high concentrations of DKK1 were related to abnormal IMT in these patients. Our results are in accordance with previous reports showing the relationship between vascular disease and DKK1 in other populations. Patients with cerebrovascular disease have higher serum DKK1 levels compared with control subjects [25], and serum DKK1 correlated with coronary artery calcification and atherosclerotic plaques evaluated by coronary computed tomography and coronary artery calcium scoring [17]. Previously, Ueland and colleagues [26] demonstrated that DKK1 expression is enhanced in advanced carotid plaques and that DKK1 is a novel mediator in platelet-mediated endothelial cell activation, which could occur within the atherosclerotic lesions. Moreover, current data clearly demonstrate a novel role for Wnt proteins in the proliferation, migration, and survival of smooth muscle cells (SMCs) [27]. Regarding this, higher levels of DKK1 are present in advanced carotid plaques and DKK1 promotes platelet-mediated activation of endothelial cells. Thus, it has been proposed that this could lead to enhanced inflammation in the atherosclerotic lesion; however, it may also lead to a reduced survival, proliferation, and migration of SMCs, thereby reinforcing the potential complexity of the participation of Wnt proteins in atherosclerosis [27].

In contrast to our findings, DKK1 concentrations were negatively associated with atherosclerotic calcified plaque in African-Americans (AAs) patients with T2DM [18]. There are several reasons that might contribute to this discrepancy. As the authors point, AAs have lower rates of vascular calcification. It has been reported lower levels of coronary artery calcified plaque relative to European Americans (EAs) despite similar or more detrimental cardiovascular risk factor profiles [28], [29]. This observation is consistent both in T2DM and subjects without diabetes [30], [31], and suggests a differential impact of CV risk factors on atherosclerosis based on ethnicity although the underlying causes of ethnic differences in vascular calcification are not well established [32]. Reinforcing these ethnic differences, AAs are at lower risk for myocardial infarction based on less coronary artery calcification, and they have significantly lower rates of myocardial infarction than EAs, provided equal access to healthcare [33], [34]. It has been suggested that AAs may be less susceptible than whites to hyperglycaemia–induced macrovascular disease [35]. Thus, these findings may explain the differences observed in the relationship between vascular disease and DKK1 between our study and the data from Register and colleagues.

Recent data suggest that the association between vascular calcification and osteoporosis is not simply a consequence of age, stressing that the co-incidence of vascular calcification with low bone activity and osteoporosis could be biologically linked. During the development of vascular calcification, there is a transition of vascular SMCs towards an osteoblast- like phenotype, which promotes the mineralization within these structures. In this process there are several players, including those related to mineral metabolism, like phosphorus, calcium or parathyroid hormone, which influences the expression of osteogenic factors [36]. There is emerging evidence suggesting that some inhibitors of the Wnt pathway, such as secreted frizzled Proteins 2 and 4 and DKK-1, may play a role linking vascular calcification and bone loss [26], [27]. AAs manifest a skeletal resistance to the effects of parathyroid hormone [37], [38] and exhibit opposite relationships between arterial calcification and serum vitamin D concentrations compared with Europeans [39]. In our opinion, these ethnic differences in the effects of calciotropic hormones on bone and vessels may indicate that differences in other pathways, as Wnt pathway, may be expected according to race. However, this hypothesis must be confirmed.

There are other differences between the study from Register and colleagues and ours, which may explain the discrepancy of results. First, the prevalence of cardiovascular disease is lower, 27%, compared to our study where a 58% of patients had previous cardiovascular disease. Second, in the study from Register and colleagues 28,3% of subjects were taking hormonal therapy while no women were on hormonal therapy in our study. Estradiol and progesterone has shown to regulate Wnt pathways in endometrial tissue [40] and brain [41], so an influence of hormonal therapy in DKK1 concentrations can not be excluded. Finally, despite the low expected incidence of arterial calcification in AAs, in the study from Register and colleagues there is a high prevalence of aortic calcification. Thus, differences between study populations apart from the ethnicity may influence the discrepancy of results.

There are no previous studies evaluating differences in serum DKK1 concentrations according to the presence of diabetes. We did not find differences in DKK1 between T2DM patients and subjects without diabetes. These results contrast with our previous data showing higher sclerostin concentrations in the same group of type 2 diabetes patients [42]. However, serum DKK1 and sclerostin have shown to be barely correlated [43], as we found in our sample (data not shown). In addition, the effects of DKK1 and sclerostin may differ due to differences in Kremen binding. While DKK1 is always an inhibitor of Wnt signaling, the effect of sclerostin is variable depending on context [44]. Unlike our previous results about sclerostin, women had higher DKK1 concentrations both in the total sample and in the T2DM group. Estradiol and progesterone have shown to take part in the regulation of the Wnt pathways in endometrial tissue [40] and brain [41], so a sex steroid-induced effect may explain gender differences in DKK1 although this hypothesis must be confirmed.

Regarding the relationship between bone metabolism and DKK1 we only found a weak correlation with BMD at lumbar spine in the total sample, and no relationship with bone turnover markers, osteoporosis or morphometric vertebral fractures (data not shown). In addition, lumbar spine BMD may be affected by aortic calcification. Our findings confirm previous data showing no relationship between DKK1 and bone turnover markers in postmenopausal osteoporosis [20]. The relationship between DKK1 and BMD is not totally established. It has been reported no relationship with volumetric BMD in Afro-American T2DM patients [18]. However, is also been described and inverse relationship between DKK1 and BMD, and higher DKK1 concentrations in patients with osteoporosis [19]. Therefore, in our opinion the data about serum DKK1 and bone metabolism are controversial and thus no clear conclusions can be drawn.

Our study has some limitations. First, the cross-sectional design does not allow to establishing a cause-effect relationship. Second, the sample size is relatively small and might affect the statistic power. Our study has also several strengths, as the evaluation of circulating serum DKK1 in Caucasian patients with T2DM for the first time, the comparison to non-diabetic subjects and the evaluation both of bone metabolism and atherosclerotic disease.

In summary, serum levels of DKK1 are elevated in Caucasian patients with T2DM presenting CVD. These findings suggest that Wnt signaling pathway is involved in vascular disease in diabetic subjects. However, the relations of DKK1 with bone metabolism are inconsistent.
