# Correction: Relationship of Dickkopf1 (DKK1) with Cardiovascular Disease and Bone Metabolism in Caucasian Type 2 Diabetes Mellitus

**DOI:** 10.1371/journal.pone.0117687

**Published:** 2015-01-27

**Authors:** The PLOS ONE Staff

The image for Fig. 1 is incorrect. Please view the correct Fig. 1 here.

**Figure 1:**
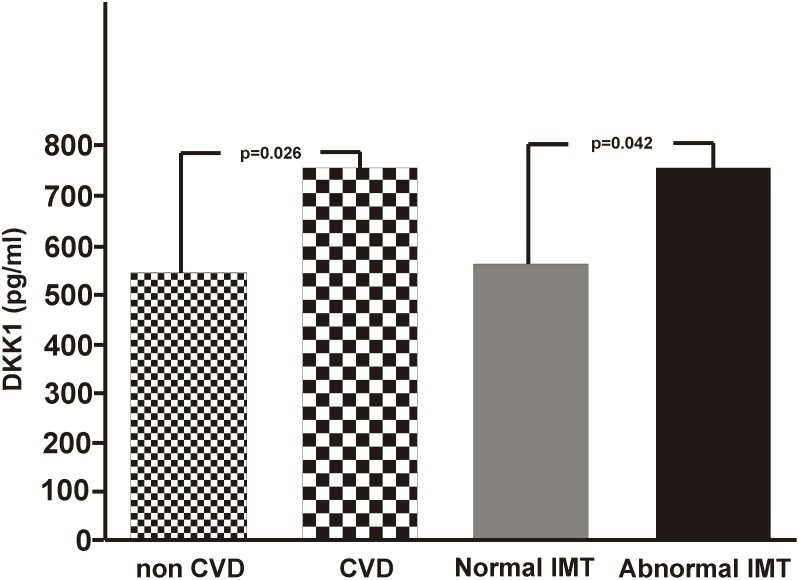
DKK1 serum levels in T2DM patients according to the presence of cardiovascular disease and abnormal intima-media thickness. Between groups differences are indicated through a bar with the P-value given above.
